# Hcp2a of APEC affects mRNA splicing and protein quality control in DF-1 cells

**DOI:** 10.1186/s12917-022-03440-z

**Published:** 2022-09-13

**Authors:** Xiangjun Song, Zhe Chen, Ziqi Li, Xiaoru Wang, Manman Hou, Ying Shao, Jian Tu, Kezong Qi

**Affiliations:** 1 Anhui Province Key Laboratory of Veterinary Pathobiology and Disease Control, College of Animal Science and Technology, Anhui Agricultural University, Hefei, 230036 PR China; 2 Anhui Province Engineering Laboratory for Animal Food Quality and Bio-safety, College of Animal Science and Technology, Anhui Agricultural University, Hefei, 230036 PR China; 3 Key Laboratory for Agri-Food Safety, School of Resource & Environment, Anhui Agricultural University, Hefei, Anhui 230036 PR China

**Keywords:** Avian pathogenic *Escherichia coli*, Type VI secretion system, Hcp, Spliceosome, Protein quality control

## Abstract

**Background:**

Bacteria deliver effector proteins into the host cell via a secretory system that can directly act on the target to cause disease. As an important pipeline structural protein of the type VI secretion system (T6SS) complex, Hcp acts together with other virulence factors in the target cell. There is growing evidence that T6SS plays a key role in the pathogenic mechanism of APEC. However, the regulatory function played by the effector protein Hcp during its interaction with host cells is not clear. Here, tandem mass tag (TMT) analysis was used to quantify the proteins affected by increased expression of Hcp2a in DF-1 cells.

**Results:**

The host response was significantly different between the overexpression and null groups at the protein level. A total of 195 differentially expressed proteins (DEPs) were detected in the overexpression group (upregulated, *n* = 144, downregulated, *n* = 51). Gene Ontology (GO) and Kyoto Encyclopedia of Genes and Genomes (KEGG) analyses were performed to predict the biological functions and pathways of differentially expressed proteins. The results showed that these DEPs were mainly enriched in RNA degradation, spliceosome, and mRNA surveillance pathways.

**Conclusions:**

This study suggests that Hcp2a, the effector protein of APEC, plays an important role in regulating mRNA splicing and protein quality control in DF-1 cells. These findings provide useful clues to elucidate the pathogenic mechanism of effector protein Hcp2a on host target cells.

**Supplementary Information:**

The online version contains supplementary material available at 10.1186/s12917-022-03440-z.

## Background

Avian Pathogenic *Escherichia coli* (APEC) is one of the most economically destructive pathogens and seriously damages the poultry industry [1]. It is mainly transmitted through the respiratory and digestive tracts and causes local or systemic infections in poultry, so-called “avian colibacillosis” [2]. Bacteria have evolved various molecular mechanisms to enhance their own colonization and invasion in the host, among which the type VI secretion system (T6SS) plays an important role by delivering various enzymes, toxins, or effectors to competing bacteria or host cells [3]. The gene cluster encoding T6SS-2 is usually overexpressed in pathogenic bacterial strains with high virulence properties and regulates bacterial pathogenicity to host cells [4]. The Hcp is one of the core components of T6SS to achieve its function as a transporter receptor and chaperone protein for effectors [5]. According to previous studies, Hcp induces cytoskeletal actin rearrangements in HBMECs in the genome of RS218, an *E. coli* K1 strain that causes meningitis [6]. A previous study from our laboratory found that Hcp2b promotes APEC colonization by affecting keratin filament expression [7]. Another study used RNA-seq to investigate the effect of infection with AE17 wild strain and *hcp*2a gene deletion strain on chicken tracheal mucosal epithelial cells [8]. However, the pathogenic mechanism of Hcp2a on protein expression in host cells remains unknown. To assess the effect of Hcp2a on eukaryotic proteome expression, we transfected eukaryotic plasmids expressing Hcp2a into DF-1 cells and examined the differentially expressed proteins in DF-1 cells by TMT (Tandem Mass Tag-Based) quantitative proteomics analysis and the dynamic changes in DF-1 cells.

## Results

### Hcp2a-induced protein changes in DF-1 cells

**Fig. 1:**
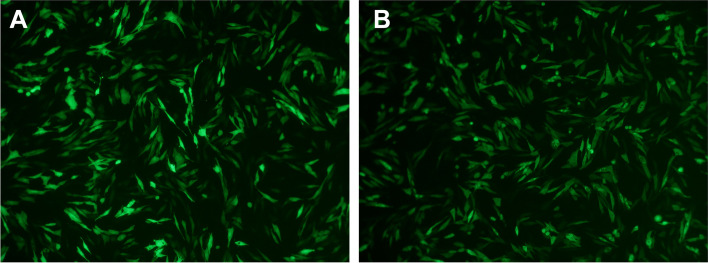
Overexpression of Hcp2a protein in DF-1 cells. Fluorescence microscopic observation of DF-1 cells at 24 h after transfection with the pEGFP-N1 (**A**) or pEGFP-N1-Hcp2a (**B**) expression vector

**Fig. 2:**
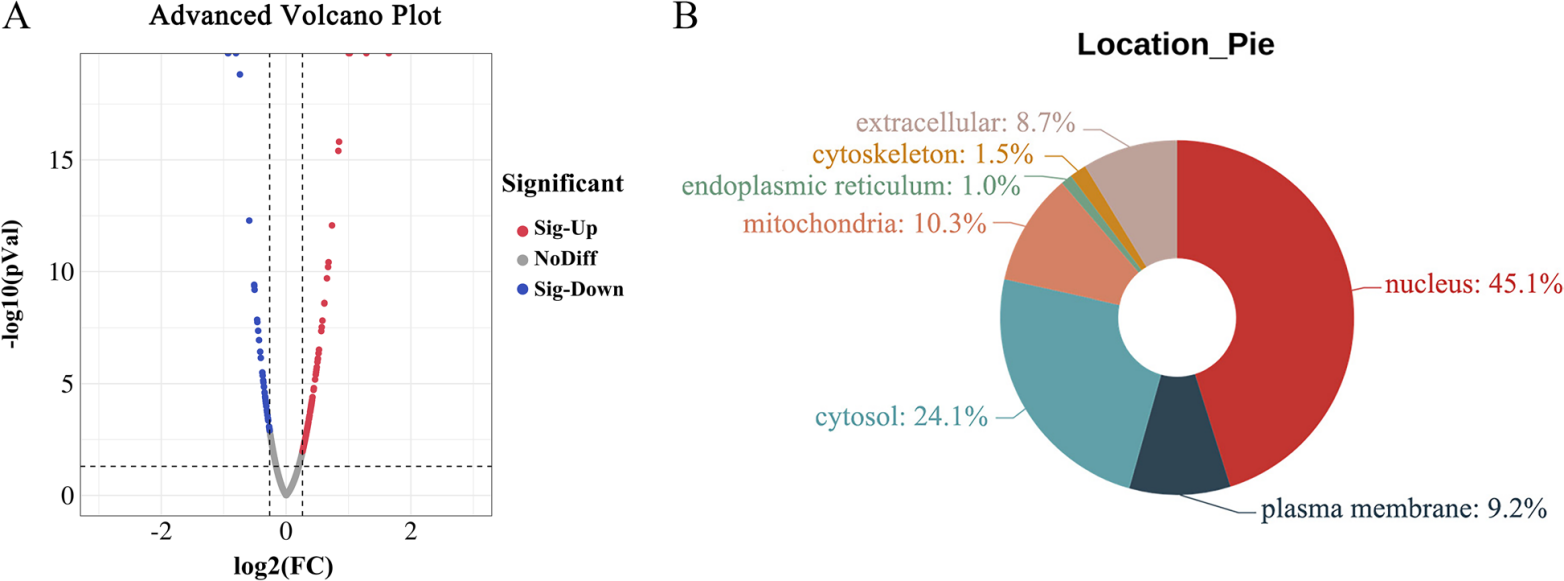
DEPs induced by Hcp2a overexpression in DF-1 cells after 24 h. **A** Volcano plot of DEPs, red dots represent significantly upregulated proteins and blue dots represent significantly downregulated proteins. **B** Subcellular localization of DEPs

In this study, Hcp2a protein was overexpressed in DF-1 cells by electroporation transfection (Fig. 1B), and the empty vector pEGFP-N1 was used as a control (Fig. 1A). To fully investigate the effect of Hcp2a on the protein level of DF-1 cells, TMT labeling and LC-MS/MS analysis were performed. By comparing the obtained peptides with the chicken reference database, we identified 4,426 proteins. A total of 195 DEPs (144 upregulated and 51 downregulated) were identified according to our differentially expressed protein identification criteria, comparing the proteomic profiles of overexpressed and null control cells (Supplementary Table S1). These results were visualized by constructing volcano maps of the differential proteins (Fig. 2A). The subcellular localization of DEPs was analyzed using WoLF PSORT, and the results showed that DEPs were widely distributed in the nucleus, cytosol, plasma membrane, and extracellular matrix (Fig. 2B).

### Functional classification of DEPs

**Fig. 3:**
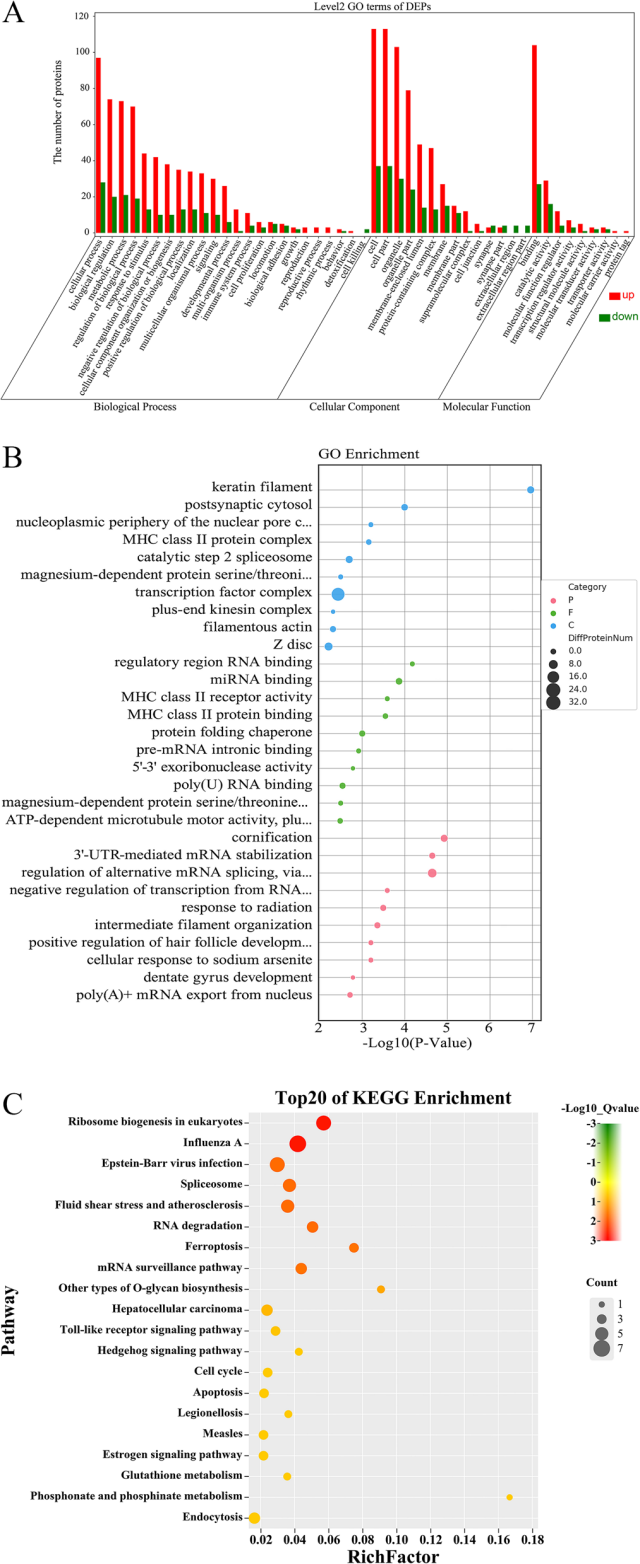
Enrichment analysis of GO and KEGG on DEPs. **A** GO annotation with upregulated and downregulated proteins in level 2. **B** Top 30 enriched GO categories in biological processes, molecular functions, and cellular components at 24 h. **C** KEGG enrichment analysis of the DEPs

We used GO database analysis to reveal the functional significance of these identified proteins and classified them according to the Gene Ontology annotation; each term was finally grouped into secondary definitions summarized by biological process (BP), molecular function (MF), and cellular component (CC) (Fig. 3A, Supplementary Table S2). The BP is mainly divided into cellular process and biological regulation. For MF, binding is the main functional class of the identified proteins. The CC was mainly distributed in the organelle part and the cell part.

For each category, the enrichment of differentially expressed proteins for all identified proteins was examined by Fisher´s exact test. The GO terms protein enrichment analysis revealed that 3'-UTR-mediated mRNA stabilization and regulation of alternative mRNA splicing via spliceosome is the main biological process class, whereas protein folding chaperone and miRNA binding is the main molecular function class, and the transcription factor complex is the main cellular formation class (Fig. 3B). This result is consistent with GO annotation, suggesting that Hcp2a plays a substantial role in regulating DF-1 cellular mRNAs.

### Analyses of signaling pathways significantly affected by DEPs

Subsequently, we performed KEGG analysis to identify the pathways involved in Hcp2a-induced differentially expressed proteins. The results showed that the enriched pathways were mainly focused on RNA degradation, spliceosome, and mRNA surveillance pathways [9–11] (Fig. 3C, Supplementary Table S3). Notably, among the proteins involved in these pathways, heterogeneous nuclear ribonucleoprotein A1 (HNRNPA1) and heterogeneous nuclear ribonucleoprotein M (HNRNPM) were significantly upregulated. The HNRNP family is a group of RNA-binding proteins that can inhibit splicing and promote alternative splicing by directly antagonizing the recognition of splice sites.

### PPI network construction for DEPs

**Fig. 4:**
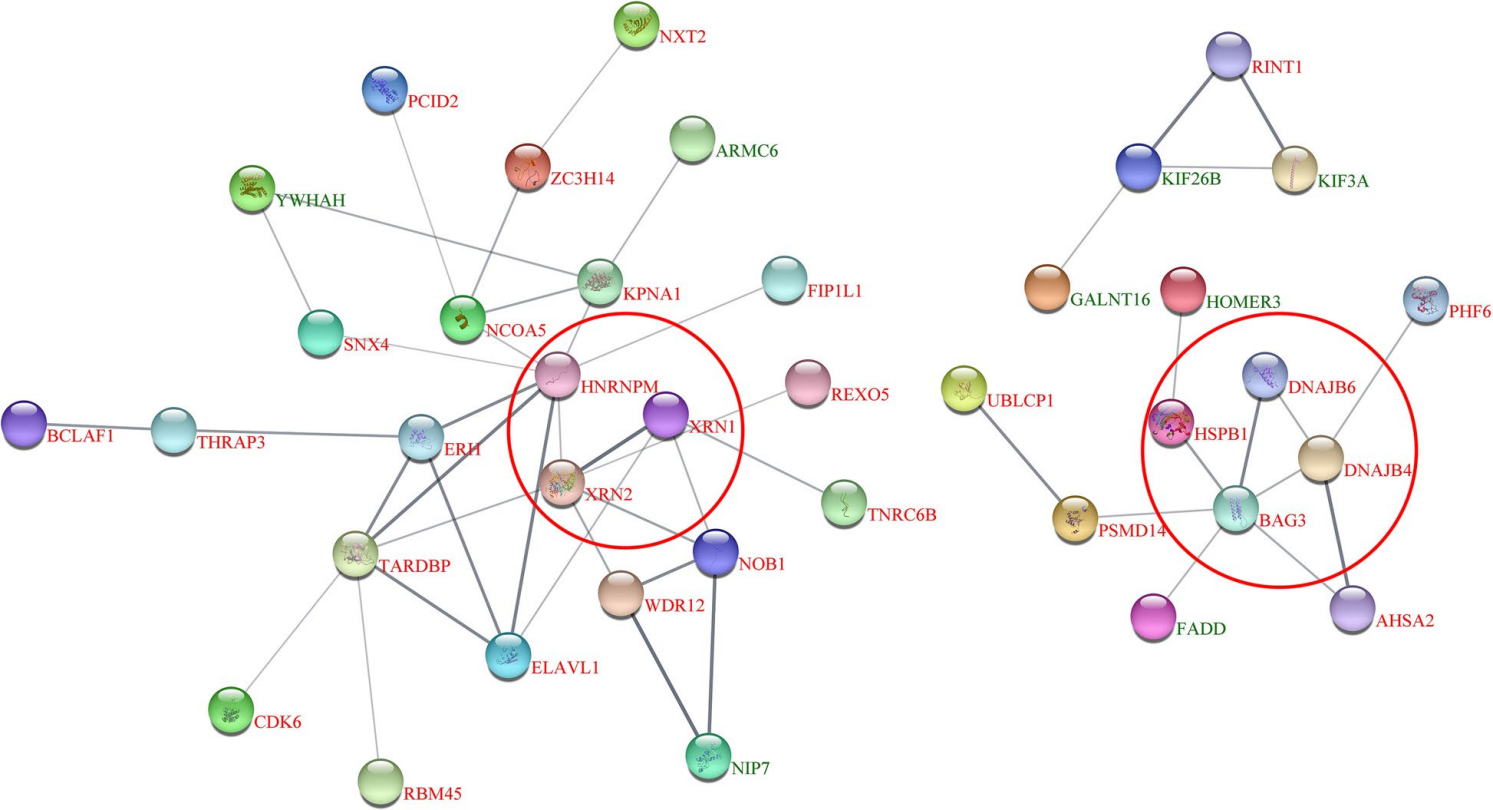
Protein-protein interaction network analysis of differentially expressed proteins

To explore the key protein interaction network induced by Hcp2a overexpression in DF-1 cells, we analyzed 195 DEPs in this study, using STRING (Fig. 4). The PPI network consisted of 47 proteins and was divided into two clusters, namely the BAG3-DNAJs-HSPB1 and HNRNPM-XRN1-XRN2 clusters. The proteins of the two clusters were enriched in the mRNA splicing and RNA degradation pathways, respectively. These results further suggest that two clusters, centered on BAG3 and HNRNPM, play important roles in DF-1 cell mRNA splicing and protein quality control.

## Discussion

The type VI secretion system is an important virulence factor for APEC and plays an important role in APEC infection by secreting a variety of effector proteins involved in the modification and manipulation of various cellular processes [12]. The Hcp is regarded as a marker of functional T6SS [5], whereas the pathogenic mechanism of Hcp2a in host cells is not clear. To investigate the profound changes of Hcp2a on host cells, we performed a comprehensive analysis of protein expression alterations in DF-1 cells in response to Hcp2a overexpression, using the TMT approach. Our data showed that a total of 195 differential proteins were detected in DF-1 cells after Hcp2a overexpression, of which 144 were significantly upregulated and 51 were downregulated. The GO enrichment analysis revealed that DEPs were closely associated with the regulation of alternative mRNA splicing via spliceosome and protein folding chaperone. The KEGG enrichment analysis showed that DEPs were significantly enriched in RNA degradation and spliceosome.

Eukaryotes exhibit an active RNA degradation system, and RNA molecules defective in protein processing, folding, and assembly are rapidly recognized and degraded. In this study, several proteins involved in the RNA degradation and spliceosome pathways, such as Pat1, XRN1, XRN2, Lsm5, Slu7, HNRNPA1, and HNRNPM, were upregulated in DF-1 cells after Hcp2a overexpression. Lsm5 is involved in the composition of two distinct heptameric complexes: the cytoplasmic Lsm1-7 complex mediates mRNA decay by binding to the protein Pat1 [13], an interaction of central importance in the mRNA decay pathway. Subsequently, Pat1 combined with Xrn1 mediated mRNA degradation. The nucleolar Lsm2-8 complex is localized in the nucleus and works in concert with XRN2 to mediate nuclear mRNA decay [14]. In addition, Lsm2-8 acts as a chaperone of U6 spliceosome RNA and binds specifically to the 3' end of U6 snRNA. Correspondingly, the splicing protein Slu7 [15] plays an important role in 3'ss selection in the second step of splicing. Intriguingly, HNRNPA1 and HNRNPM were significantly upregulated in our study. The HnRNPA1, with the relative concentration of the must splice factor SF2, precisely selects the 5' splice site and promotes exon skipping [16, 17]. The HNRNPM is a splicing regulator that promotes CD44-variable exon skipping [18]. Previous studies have confirmed that alternative 3′ splice sites and exon skipping/inclusion (ESI) are the main alternative splicing (AS) events in chicken primary immune organs under systemic APEC infection [19]. Therefore, we hypothesized that Hcp2a plays an important role in AS events in DF-1 cells. To further identify key protein interactions in DF-1 cells after Hcp2a overexpression, we performed PPI analysis and identified an interaction between BAG3-DNAJs-HSPB1. Previous studies have shown that when the level of DNAJs in cells is increased and the ATPase is over-stimulated, substrate capture is prevented, thereby forming erroneous protein aggregates [20]. In this process, BAG-type NEFs can link the release of chaperone substrates to downstream cellular processes that determine the fate of the substrates [21]. Driven by increased expression of BAG3, misfolded aggregation-prone proteins are recognized and targeted by molecular chaperones HSPB8 and HSP70, with direct or indirect involvement of other molecular chaperones (e.g., HSPB1) [22]. The BAG3 has been shown to recruit the autophagy receptor p62/SQSTM1, which binds to the autophagy marker protein LC3 for retrograde transport along microtubules in a Kinesin/dynein (motor protein on microtubules)-dependent manner to the microtubule organizing center (MTOC), where it is subsequently assembled into protein structures called “aggresomes” [23, 24]. Notably, proteomic analysis in this study showed that the expression of four autophagy markers (BAG3, MAP 1LC3B2, KIF15, and DNAL4) was significantly upregulated in cells after Hcp2a overexpression. In view of the above results, we speculate that Hcp2a may disrupt intracellular protein homeostasis in DF-1 cells through the upregulation of DNAJs.

## Conclusions

In summary, we identified proteins closely associated with Hcp2a overexpression in DF-1 cells, using the TMT technology. Most of the proteins were enriched in RNA degradation, alternative splicing spliceosomes, and intracellular protein quantity control pathways, suggesting that Hcp2a plays an important role in regulating mRNA splicing and protein quantity control in eukaryotic cells. These findings provide useful clues to elucidate the pathogenic mechanism of effector protein Hcp2a on host target cells.

## Methods

### Cell culture

Chicken embryonic fibroblasts (DF-1) used in this study were maintained by the Anhui Provincial Key Laboratory of Veterinary Pathobiology and Disease Prevention and Control. Cells were maintained in DMEM/F-12 supplemented with 10% fetal bovine serum (Gibco, Gaithersburg, MD, USA) and 1% antibiotic solution (100 U/mL penicillin and 100 μg/mL streptomycin) at 37°C under an atmosphere of 5% CO_2_.

### Plasmid construction and transfection

All enzymes used for the cloning procedure were purchased from TAKARA (Dalian, China). Plasmid pEGFP-N1 was stored in our laboratory; primers were designed by Primer Premier 5.0. The *hcp*2a gene sequence (GenBank accession number: CP000468.1) was adjusted with reference to chicken codons preferences, and the fragment was cloned into the *Xho* I and *EcoR* I sites in the pEGFP-N1 vector; the recombinant plasmid was named pEGFP-N1-Hcp2a. The constructed recombinant expression plasmid was confirmed by restriction digestion analysis and DNA sequencing. The DF-1 cells were cultured to 80% confluence in six-well plates, and then pEGFP-N1-Hcp2a was mixed with the cell suspension in an ice bath, and transfection was completed by electroporation. The pEGFP-N1 was also transfected as a control and observed using fluorescence microscopy.

### Labeling of protein tandem mass spectrometry tags

Cell samples were collected 24 hours after transfection and washed three times in sterile PBS. The samples were treated with lysis buffer (8M urea, 1% protease inhibitors), sonicated on ice, and centrifuged at 12,000g for 10 min at 4°C; the supernatant was collected, and the samples were divided and stored at -80°C. The protein concentration in the extracts was determined using the BCA protein quantification kit, and the samples were separated by SDS-PAGE. Enzymatic digestion was performed using the filter-aided proteome preparation (FASP) method. Then, 100 μg of peptides were taken from each sample and labeled with the TMT isotope labeling kit (Thermo Fischer Scientific co. US) according to the instructions. The labeled peptides from each group were mixed and graded using an Agilent 1260 infinity II HPLC system. Briefly, the column was first equilibrated with 10 mM HCOONH_4_, 5% ACN (pH 10.0), followed by loading of the mixed labeled samples onto the column for separation. The column was eluted by gradient increasing buffer (10 mM HCOONH_4_, 85% CAN, pH 10.0) to separate the peptides at a flow rate of 1 mL/min. During elution, the absorbance value at 214 nm was monitored, and the eluted fractions were collected at 1-min interval; the samples were lyophilized and re-dissolved with 0.1% FA.

### LC-MS/MS analysis

Each sample was separated using an Easy-nLC 1200 nanoflow HPLC system (Thermo Fisher Scientific). Buffer A was 0.1% formic acid aqueous solution, and liquid B was 0.1% formic acid acetonitrile aqueous solution (acetonitrile was 80%). The chromatographic column was equilibrated with 100% Buffer A. The samples were separated by the autosampler onto an analytical dwelling (Thermo Fisher Scientific, Acclaim PepMap RSLC 50um X 15cm, nano viper, P/N164943) for 90 min at a flow rate of 300 nL/min.

The samples were separated by chromatography and then analyzed by mass spectrometry using a Q Exactive Plus mass spectrometer. The mass-to-charge ratio of peptides and peptide fragments was collected according to the following method: 10 fragmentation profiles (MS2 scan) were collected after each full scan. The MS2 activation type was HCD, the isolation window was 2 m/z, the secondary mass spectrometry resolution was 3,500, the microscan number was 1, the secondary maximum IT was 45ms, the normalized collision energy was 30 eV.

### Data analysis

The raw mapping files generated by Q Exactive Plus were submitted to the MASCOT2.6 server for database search using the Proteome Discoverer 2.1 (Thermo Fisher Scientific) software. The database used for this project was UniProt Gallus gallus (http://www.uniprot.org). The data were screened according to the criterion of FDR < 0.01.

### Bioinformatics analysis

The screening criteria for differential proteins were ploidy change > 1.2 and < 0.83, and *P* value < 0.05. Advanced volcano and heatmap plots were generated using the OmicStudio tools at https://www.omicstudio.cn/ tool.

The DEPs were annotated for functional analysis using the Gene Ontology (GO) and Kyoto Encyclopedia of Genes and Genomes (KEGG) databases, respectively. Statistical significance was determined by Fisher's two-tailed exact test; GO terms and KEGG pathways with corrected *P* values below 0.05 were considered significant. The protein-protein interaction (PPI) networks of DEPs were constructed by STRING and visualized in Cytoscape. Subcellular structure localization prediction and classification statistics of differentially expressed proteins were performed using WoLF PSORT.

### Statistical analysis

For all experiments, three independent replicates were analyzed. Statistical analysis was performed using the GraphPad Prism5 software (GraphPad, USA) for Student's t-test, and *P* values below 0.05 were considered significant.

## Supplementary Information

**Additional file 1: Supplementary Table S1.** The differentially expressed proteins comparing the experimental group and control group.

**Additional file 2: Supplementary Table S2.** GO and GO enrichment analysis of the DEPs.

**Additional file 3: Supplementary Table S3.** KO categorization and KEGG enrichment analysis of the DEPs.

## Data Availability

All data generated or analyzed during this study are included in this published article [and its supplementary information files].
